# Quantifying contributions of natural variability and anthropogenic forcings on increased fire weather risk over the western United States

**DOI:** 10.1073/pnas.2111875118

**Published:** 2021-11-01

**Authors:** Yizhou Zhuang, Rong Fu, Benjamin D. Santer, Robert E. Dickinson, Alex Hall

**Affiliations:** ^a^Department of Atmospheric and Oceanic Sciences, University of California, Los Angeles, CA 90095;; ^b^Program for Climate Model Diagnosis and Intercomparison, Lawrence Livermore National Laboratory, Livermore, CA 94550

**Keywords:** western United States, fire weather, attribution, atmospheric circulation, anthropogenic warming

## Abstract

Previous studies have identified a recent increase in wildfire activity in the western United States (WUS). However, the extent to which this trend is due to weather pattern changes dominated by natural variability versus anthropogenic warming has been unclear. Using an ensemble constructed flow analogue approach, we have employed observations to estimate vapor pressure deficit (VPD), the leading meteorological variable that controls wildfires, associated with different atmospheric circulation patterns. Our results show that for the period 1979 to 2020, variation in the atmospheric circulation explains, on average, only 32% of the observed VPD trend of 0.48 ± 0.25 hPa/decade (95% CI) over the WUS during the warm season (May to September). The remaining 68% of the upward VPD trend is likely due to anthropogenic warming. The ensemble simulations of climate models participating in the sixth phase of the Coupled Model Intercomparison Project suggest that anthropogenic forcing explains an even larger fraction of the observed VPD trend (88%) for the same period and region. These models and observational estimates likely provide a lower and an upper bound on the true impact of anthropogenic warming on the VPD trend over the WUS. During August 2020, when the August Complex “Gigafire” occurred in the WUS, anthropogenic warming likely explains 50% of the unprecedented high VPD anomalies.

## Significance

The western United States (WUS) has experienced a rapid increase of fire weather (as indicated by vapor pressure deficit, VPD) in recent decades, especially in the warm season. However, the extent to which an increase of VPD is due to natural variability or anthropogenic warming has been unclear. Our observation-based estimate suggests ∼one-third of the VPD trend is attributable to natural variability of atmospheric circulation, whereas ∼two-thirds is explained by anthropogenic warming. In addition, climate models attribute ∼90% of the VPD trend to anthropogenic warming. Both estimates suggest that anthropogenic warming is the main cause for increasing fire weather and provide a likely range for the true anthropogenic contribution to the WUS trend in VPD.

**Fig. 1.**
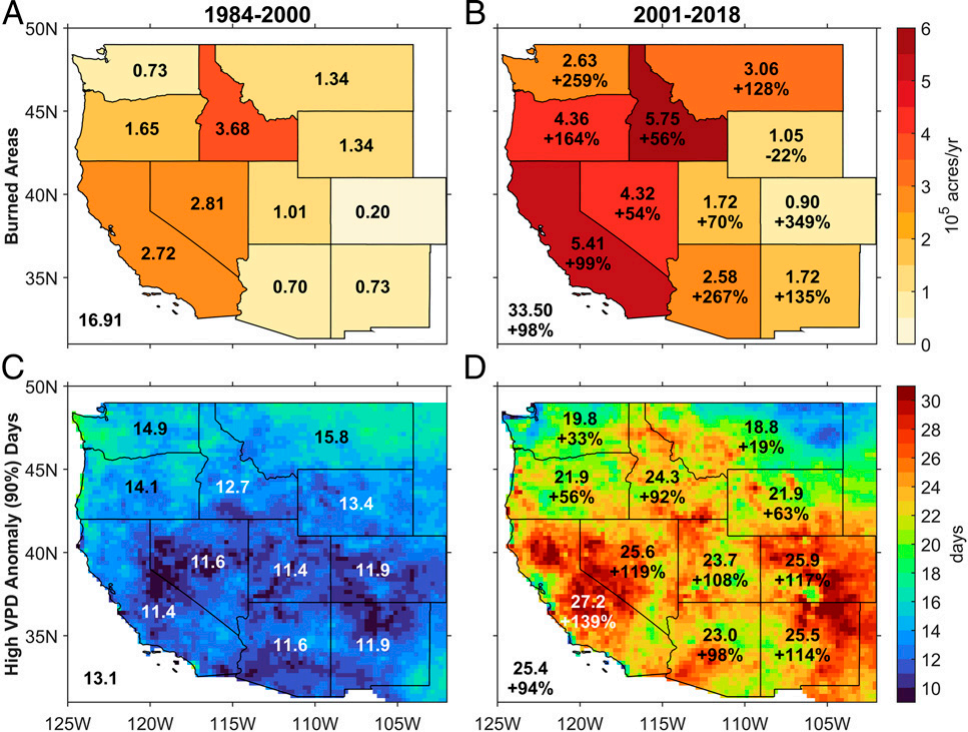
(*A*) Annual mean burned areas (10^5^ acres/yr) in the warm season during the period 1984 to 2000. Results for the average of each state are given by shading and with a numerical value. The averaged burned areas over the whole WUS are shown in the *Lower Left* corners. (*B*) Same as *A* but for the period of 2001 to 2018. The percentage changes of burned areas relative to those of the 1984 to 2000 period are shown below the annual mean burned areas. (*C*) Average days with high VPD (percentile VPD′ over 90% in a year) for the 1984 to 2000 period. (*D*) Same as *B* but for the averaged days with high VPD.

The western United States (WUS) is prone to large wildfires, over 90% of which occur in the warm season (May to September) according to the Monitoring Trends in Burn Severity (MTBS) database (1). The year 2020 was a record-breaking fire season in the history of the WUS, especially in the coastal states of California, Oregon, and Washington. Many recent studies of fire behavior in the WUS have indicated warm season increases in the area burned by fires, fire frequency and intensity, and fire season length (2–12). Analysis of the MTBS data in Fig. 1 *A* and *B* shows that the average warm season burned area in the WUS during 2001 to 2018 was about 3.35 million acres, nearly double (+98%) that of the previous period of 1984 to 2000 (1.69 million acres). According to the National Interagency Fire Center (NIFC) report, the area burned by wildfire during the 2020 warm season reached 8.8 million acres (13, 14), more than five times the average during 1984 to 2000. This rapid increase in burned area has been observed across most of the WUS except in Wyoming. It has been linked to more extreme fire weather risk, largely due to high vapor pressure deficit (VPD) (10, 15, 16). In the warm season, the number of days per year with high VPD (defined as days with VPD larger than the 90th percentile value of VPD in the climatological period of 1979 to 2010) increased by 94% during 2001 to 2018 relative to 1984 to 2000 (Fig. 1 *C* and *D*).

Many factors and their complex interactions can contribute to increased fire activity. In addition to an increase in VPD or evaporative demand due to warming, fire behavior is also affected by ignition sources (17), forest management (18), tree mortality from bark beetles (19), earlier and reduced springtime snowmelt (7), reduced summer precipitation (20), cloud shading (21), vegetation cover (22), fog frequency (23), live fuel moisture content (23–25), and increase in fire-prone wind patterns (26, 27). Recently, there has been intense interest in the issue of how anthropogenic warming may impact fire behavior. Several studies have used climate model simulations to assess the impact of anthropogenic forcing on increased fire activity in the WUS (10) and in other regions (28, 29). The contribution of anthropogenic climate change is often estimated by the linear trend or long-term low-pass filtered time series of the fire indices. The contribution of atmospheric internal variability is approximated by considering the detrended fire index time series (16, 30) or by comparing historical simulations with realistic anthropogenic forcings to simulations with natural climate forcings only (28).

The VPD at synoptic to decadal time scales is closely related to atmospheric circulation patterns (31–34). Winds from hot inland areas and subsidence associated with high surface pressure systems generate hot and dry air, leading to high VPD values. However, few studies have evaluated the influence of natural internal climate variability on multidecadal changes in VPD. This is in part because of the difficulty of partitioning observed temporal variations into internally generated and externally forced components (35). Partitioning these components is more straightforward in climate models. Large initial condition ensembles are particularly useful for this purpose (36). One problem, however, is that many models may inadequately represent regional patterns of internal variability, especially over the WUS (37). Using climate model simulations to estimate the impact of natural variability of the atmospheric circulation on VPD is therefore challenging and subject to large uncertainties.

As a consequence, it has been unclear whether the observed change in VPD since 1979 exceeds the VPD change that can be explained by internal variability alone. To address this issue, and to better quantify the relative contributions of internal variability and external forcing (particularly anthropogenic forcing) to the observed increase in fire weather in the WUS, we consider an observation-based flow analogue approach (38–40). This approach characterizes VPD values based on their distribution for a given atmospheric circulation pattern (e.g., geopotential height at 500 hPa, Z500) constructed from a suite of similar circulation patterns during a climatological period (e.g., 1979 to 2010).

Different flow analogue approaches have been reported in the literature. Our analysis shows that the choice of approach and the choice of observational dataset (the reanalyses listed in *SI Appendix*, Table S1) can affect our flow analogue estimates. This is why we introduce an ensemble constructed flow analogue scheme. In this approach, multiple analogue schemes are constructed. Their interquartile range (IQR) is used to account for uncertainties in analogue VPD estimates arising from the choice of approach and observational dataset (see *Methods*). In addition, we also evaluate VPD trends in multimodel ensembles of simulations provided by the sixth phase of the Coupled Model Intercomparison Project (CMIP6; reference *SI Appendix*, Table S2). Our analysis of CMIP6 simulations yields a model-based estimate of the forced component of VPD changes, thus providing an independent check on our observational attribution of VPD trends.

## Historical Trends of Fire Weather Risk.

**Fig. 2.**
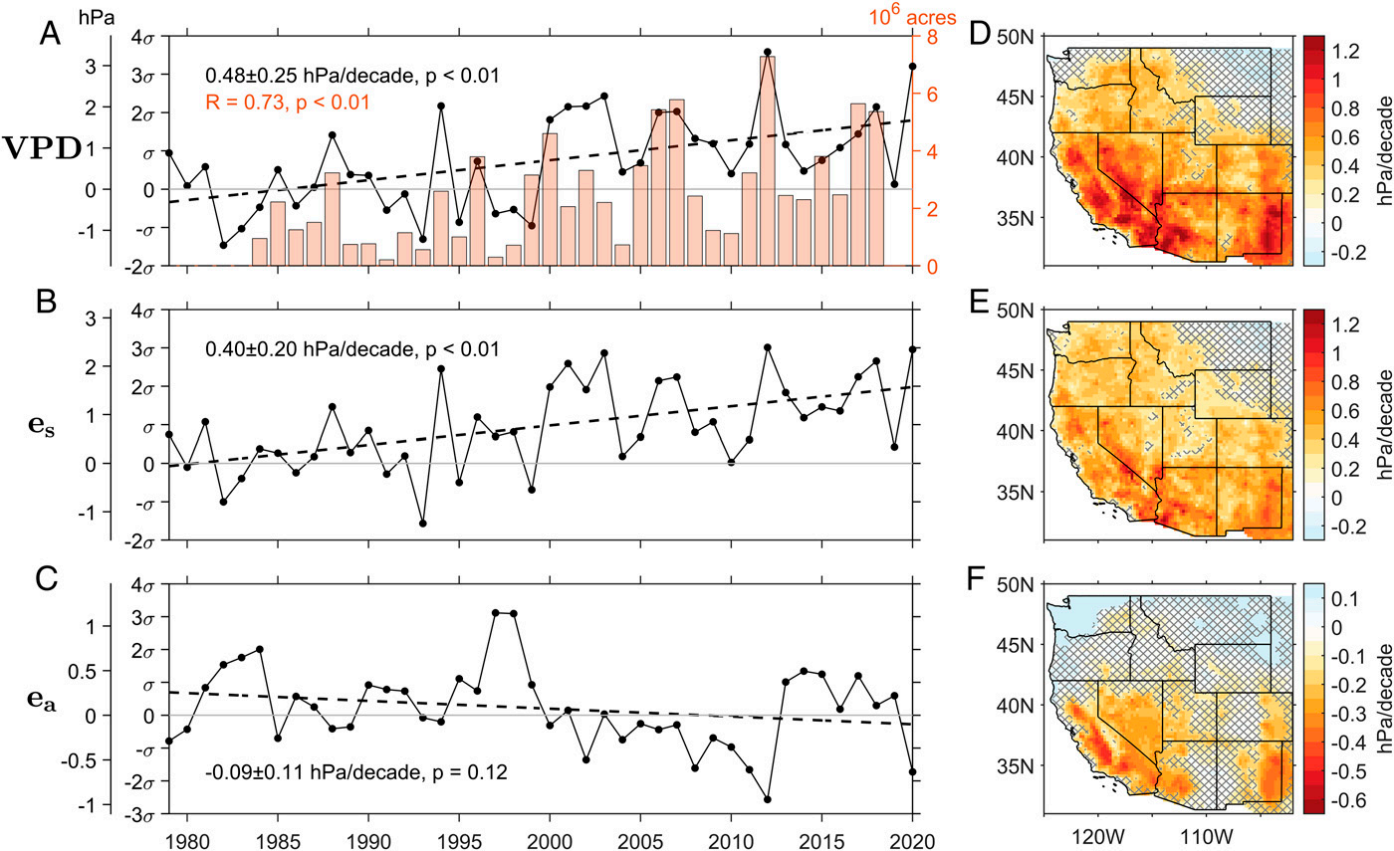
(*A*) Average time series of VPD′ from the gridMET dataset (solid line) and burned areas from the MTBS dataset (bars) for all warm season days. The VPD′ trend is the slope of the regressed line (dashed line) of the time series for all available years (1979 to 2020). The VPD′ trend for the shorter period 1984 to 2018 shows a similar result (*SI Appendix*, Fig. S1). (*B* and *C*) Same as *A* but for time series of *e*_s_ and *e*_a_. (*D–F*) Trend map of these anomalies for the WUS (all warm season days). The absence of hatching denotes regions where the trends are significant at the *P* < 0.05 level.

How has the WUS warm season fire weather risk (as represented by VPD) increased since the beginning of the satellite era in 1979? Previous studies have already shown an increase of VPD over a large area of the United States (15, 16, 32). For example, Abatzoglou and Williams (10) estimated the VPD trend over 1979 to 2015 to be 1.73 σ per 37 y (0.47 σ/decade). Here, we extend the analysis period from 2015 to 2020 using gridded surface meteorological (gridMET) (41) observations and show the linear trend in the time series of warm season mean VPD anomaly for the WUS (VPD′; *Methods*). Fig. 2*A* indicates that the warm season mean VPD′ over the WUS has increased significantly (*P* < 0.01) by 0.48 ± 0.25 hPa/decade (95% CI). After normalizing the trend by the SD (σ = 0.93 hPa) of the detrended VPD (see *Methods*) during the climatological period of 1979 to 2010, it is equivalent to 0.52 ± 0.27 σ/decade. This trend is close to the previously estimated VPD trend during 1979 to 2015 (10). The trend of increasing VPD is significant across most of the WUS, except for the northeastern WUS and part of Washington state (Fig. 2*D*). Further analysis reveals that these VPD trends are generally robust to different choices of method used for estimating the slope of a regression line (*SI Appendix*, Table S3).

Fig. 2*A* also shows that the burned area in the warm season generally follows both the VPD trend and the variations in VPD on interannual to decadal time scales. The correlation coefficient between the burned area and VPD′ time series is 0.73 (*P* < 0.01). This indicates that VPD is the leading climatic control on the burned area over the WUS. Strong functional relationships between VPD and the burned area have been found in United States and other regions of the world (10, 15, 16, 28, 42). VPD′ associated with large fire events, defined by VPD′ averaged within the areas and during the days of the large fires, are systematically higher than those of all warm season days by about 3 hPa on average (*SI Appendix*, Fig. S1). The former shows a similar increase trend to the latter.

To quantify the contributions of surface warming and drying to the VPD trend over 1979 to 2020, we evaluate the time series of saturated vapor pressure (*e*_s_; Fig. 2*B*) and actual vapor pressure (*e*_a_; Fig. 2*C*) of the surface air. Fig. 2*B* shows a significant (*P* < 0.01) trend of increasing *e*_s_ at a rate of 0.40 ± 0.20 hPa/decade (0.50 ± 0.25 σ/decade). In contrast, *e*_a_ decreases but does not show a significant negative trend (*P* = 0.12; Fig. 2*C*). Overall, the increase in *e*_s_ explains 82% of the total VPD trend, indicating that the increase in VPD over the WUS is largely due to warming (increase of *e*_s_; Fig. 2 *B* and *E*). This is generally consistent with the findings of previous studies (10). The spatial distributions of the *e*_s_ and VPD trends are very similar (Fig. 2 *D* and *E*); the drying effect represented by the decrease of *e*_a_ accounts for 18% of the trend (Fig. 2*C*) and is only significant over parts of California, Nevada, and the Southwest (Fig. 2*F*).

## Contribution of Atmospheric Circulation Changes and Anthropogenic Warming to Increasing Fire Weather Risk.

The relationship between hot and dry conditions and large-scale atmospheric circulation is well known. For example, Crimmins (31) found that 80% of the extreme fire weather days during late spring to early summer in the US Southwest were linked to the southwesterlies and anomalous high pressure systems over that region. While such a general characterization captures the averaged anomalous atmospheric circulation pattern associated with high VPD (*SI Appendix*, Fig. S2), there is a significant variation in the location, shape, and strength of the anomalous high associated with high VPD in different states of the WUS (*SI Appendix*, Fig. S3).

To quantify the contribution of the atmospheric circulation changes to the observed changes of VPD′, we apply an ensemble constructed flow analogue method modified from previous flow analogue or dynamical adjustment approaches (38, 43). For simplicity, this method is referred to as the analogue method, and the estimated VPD′ associated with atmospheric circulation is hereafter referred to as the analogue VPD′. Full details are provided in the *Methods* section. We then determine the fraction of the observed increase in VPD in recent decades can be explained by a more frequent occurrence of circulation patterns that favor high VPD, that is, by the analogue VPD′. The underlying assumption here is that any change in the frequency of a “high VPD” atmospheric circulation pattern is due to internal variability alone. If the VPD associated with a specific circulation pattern is systematically higher in recent decades than in the past, then such systematic increases in VPD are likely due to anthropogenic warming and associated thermodynamic feedbacks, particularly if they are consistent with the VPD changes simulated by global climate models in response to anthropogenic forcing.

Our results show that the daily analogue VPD′ explains a large fraction of the total variance of the observed VPD′ averaged over the WUS for all warm season days during 1979 to 2020 (*R*^2^ = 77%), indicating that the analogue method successfully captures the influence of synoptic variations in circulation patterns on VPD.

**Fig. 3.**
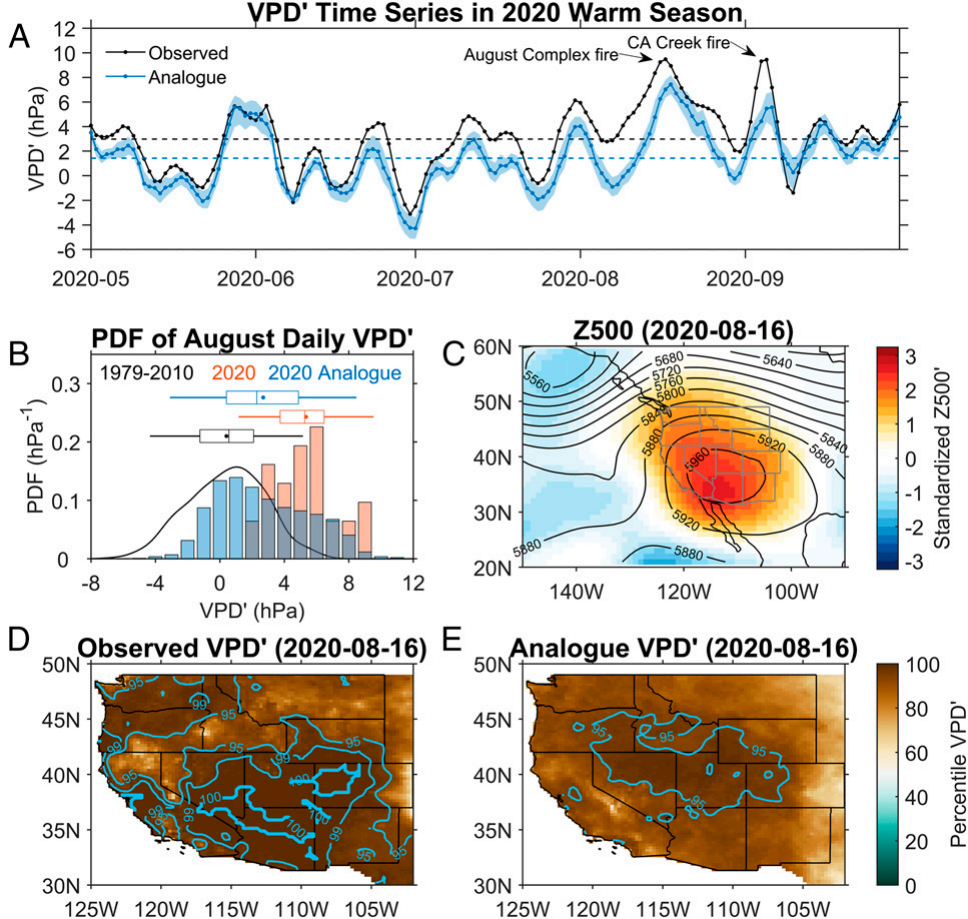
(*A*) VPD′ time series in 2020 warm season over the WUS from both observations (black line) and analogues (blue line for mean analogue; shading for IQR). Starting days of the August Complex fire and California Creek fire are labeled. Dashed horizontal lines are the warm season mean values. (*B*) PDF of August VPD′ for the observations from the climatological period of 1979 to 2010 (black curve), 2020 observations (red bars, shaded dark gray where they overlap with blue bars), and 2020 analogues (blue bars). The three vertical lines in each box plot represent the 25th, 50th, and 75th percentiles, the dot represents the mean value, and the whiskers extend to two SDs from the mean. (*C*) Map of Z500 (contours) and its standardized anomalies relative to 1979 to 2010 climatology (shading) averaged over four reanalysis datasets (the fifth generation of the European Centre for Medium-Range Weather Forecasts [ECMWF] atmospheric reanalysis [ERA5], the Modern Era Retrospective analysis for Research and Applications version 2 [MERRA-2], the National Centers for Environmental Prediction [NCEP] Climate Forecast System Reanalysis [CFSR], and the Japanese 55-y Reanalysis [JRA55]) on August 16, 2020, the start date of the August Complex fire. (*D*) Percentile VPD map on the same date as *C*, overlaid with the 95, 99, and 100% contours. (*E*) Same as *D* but for constructed analogue VPD map.

Fig. 3*A* shows the time series of the observed VPD′ compared to that of the VPD′ expected from the atmospheric circulation (i.e., the analogue VPD′) during the warm season of 2020. The 2020 warm season started with relatively mild weather conditions from May to early July. The observed VPD′ closely matches the analogue VPD′ during this period, suggesting that the observed VPD′ was mainly influenced by the variation of the atmospheric circulation. After early July, however, the observed VPD′ is higher than the analogue VPD′. This difference between observed VPD′ and analogue VPD′ is especially pronounced for the two extreme VPD′ spikes during the August Complex “Gigafire” (mid-August) and the California Creek fire (early September). These two fires were ranked No. 1 and No. 5 in California wildfire history at the time of writing (44). For August 2020 (Fig. 3*B*), the probability density function (PDF) of the observed VPD′ showed a strong shift toward high VPD′ relative to both its climatology and analogue VPD′. The mean value of the observed VPD′ in August 2020 was 4.9 hPa (∼2.1 σ) higher than that of the August climatology; the mean analogue VPD′ in August 2020 exceeded the climatological mean of the observed VPD′ by 2.3 hPa (∼1.0 σ). We conclude from this that the strong anomalous circulation condition can only explain about half of the exceptionally high VPD′ in August 2020.

On August 16, 2020 when the August Complex fire started, an extensive and strong anomalous high was centered over the Southwest and dominated the whole WUS (Fig. 3*C*); most values of VPD′ across the WUS ranked in the 99th or even the 100th percentile—that is, they were equal to or exceeded maximum VPD′ values observed in the same region (within a 31-d period centered on August 16) during the climatological period of 1979 to 2010 (Fig. 3*D*). While the analogue VPD′ on this day also show very high VPD conditions over the whole WUS, they were less extreme than the observed VPD′ (Fig. 3*E*; note that there are no contours of the 99th and 100th percentiles). In fact, averaged over the WUS, the analogue VPD′ could only account for ∼68% of the observed VPD′ for the August 16 event and even less (∼48%) for the September 4 event (Fig. 3*A*). Thus, the observed high VPD′ values during the 2020 warm fire season significantly exceeded VPD′ values that can be explained by the atmospheric circulation pattern.

**Fig. 4.**
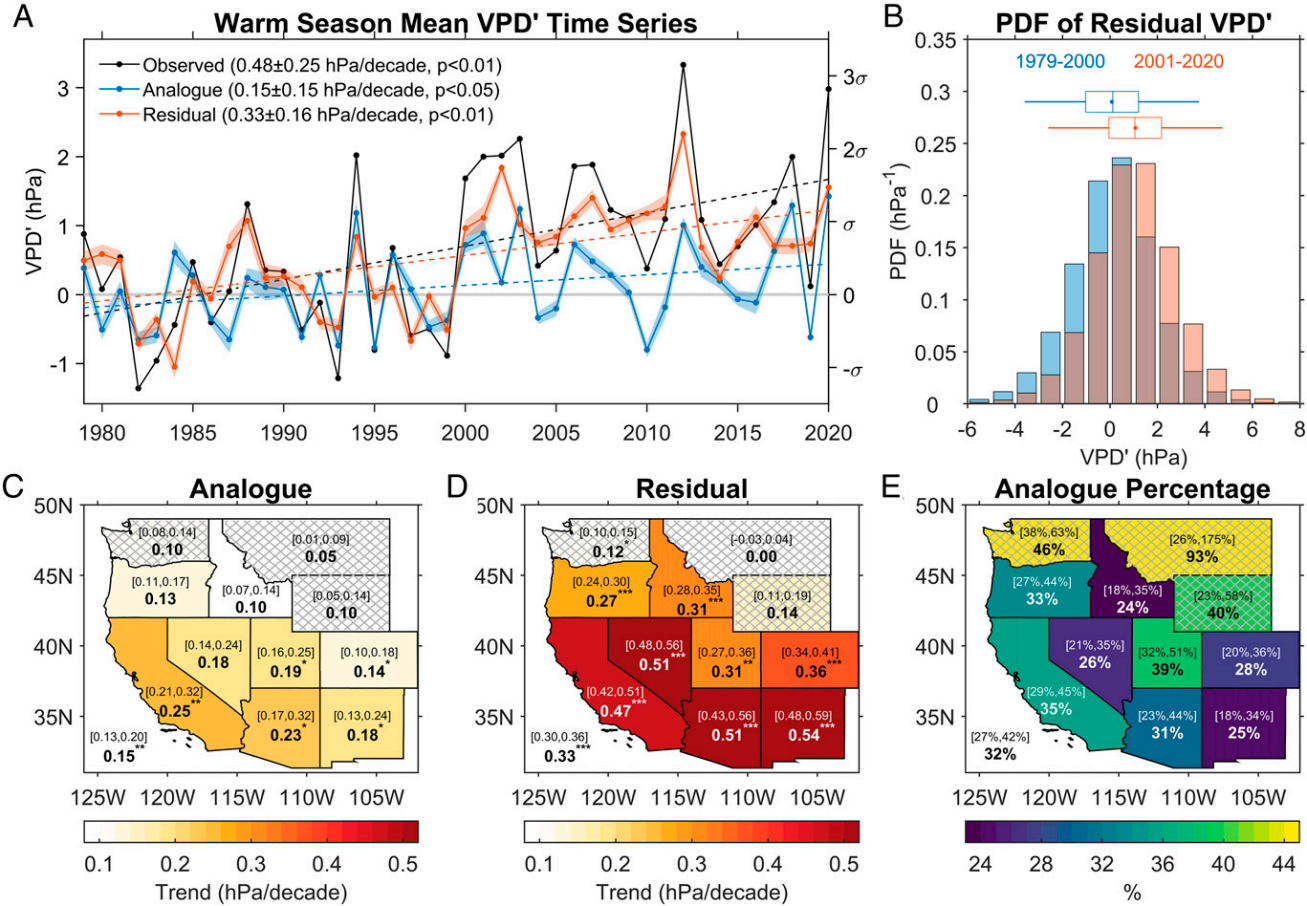
(*A*) Warm season mean VPD′ time series over the WUS from observations (black line), analogues (blue line; shading represents IQR for VPD′ from the 180 analogue schemes described in *Methods*), and residuals (observations minus analogue, red line, shading represents IQR). (*B*) PDF of the residual VPD anomalies for the periods 1979 to 2000 and 2001 to 2020, respectively, and box plots (see Fig. 3*B* for explanation). (*C*) Analogue VPD′ trend (1979 to 2020) in each state. The value shown by bold black font within each state shows the VPD trend of that state. The value shown by bold black font in the *Lower Left* corner is the VPD trend averaged over the entire WUS. One, two, or three asterisk(s) next to these trend numbers denotes trend significance at *P* < 0.1, 0.05, and 0.01, respectively. Numbers inside brackets are IQR of the trends calculated from 180 individual analogue schemes. (*D*) Same as *C* but for residual VPD′ trend (observations minus analogue). (*E*) Percentage of the analogue VPD trend relative to the observed VPD trend (IQR in brackets). Montana, Wyoming, and Washington have nonsignificant observed VPD trends at the *P* < 0.05 level (*SI Appendix*, Table S4), and the corresponding regions are therefore hatched in *C–E*.

On the interannual time scale, Fig. 4*A* shows that the analogue and observed warm season mean VPD′ time series display very similar variations (*R*^2^ = 68%). Since 2000, however, the observed VPD′ was systematically higher than the analogue VPD′. The trend of analogue warm season mean VPD′ is 0.15 ± 0.15 hPa/decade, explaining 32% of the observed VPD trend (0.48 ± 0.25 hPa/decade); the IQR of these trends for all 180 different analogue schemes (see *Methods*) is 0.13 to 0.20 hPa/decade, explaining 27 to 42% of the observed VPD trend. The residual VPD trend (observed minus analogue) is 0.33 ± 0.16 hPa/decade, explaining 68% of the observed trend; the IQR of all 180 residual trends is 0.30 to 0.36 hPa/decade, explaining 62 to 75% of the observed VPD trend.

Fig. 4*B* shows the PDF of residual VPD′. The PDF curve is basically symmetric about zero during the first two decades of our analysis period (1979 to 2000), suggesting a dominant influence of random variability of the atmospheric circulation on VPD. During the recent two decades (2001 to 2020), the mean residual VPD′ shifted to the positive side by 1.00 hPa (0.54 σ) relative to the period of 1979 to 2000. This is primarily due to a shift of +1.37 hPa (0.53 σ) in the mean observed VPD′; the shift in the mean of the analogue VPD′ (+0.38 hPa or 0.14 σ) is less than a third of that observed (*SI Appendix*, Fig. S4).

Fig. 4 *C* and *D* show the analogue and residual VPD′ trends averaged over each state in the WUS. Similar to the result for the entire WUS, most states (especially those with significant observed VPD′ trends; Fig. 1 and *SI Appendix*, Table S4) have an analogue trend that is considerably smaller than the residual trend. The trend ratio (analogue to observed) in Fig. 4*E* suggests that for the eight states with significantly positive trends for the observed VPD′, the circulation contribution ranged from 24% (Idaho) to 39% (Utah), leaving 76 to 61%, respectively, of the residual trend unexplained. Overall, these results indicate the analogue VPD′ trend associated with circulation changes can only explain about one-third of the observed VPD trend across most of the WUS.

This residual VPD′ mainly represents the thermodynamically contributed VPD′ after removing the dynamically controlled analogue VPD′. It is contributed by both thermodynamic feedbacks to the natural circulation changes, such as land surface feedbacks, and warming due to anthropogenic forcing. The relatively small residual VPD′ values prior to 2000 are presumably dominated by the thermodynamic feedbacks, whereas the systematic increase of the residual VPD′ afterward are likely contributed by anthropogenic forcings and the associated thermodynamic feedbacks.

In addition to atmospheric circulation changes, could reduced cloudiness and vegetation cover contribute to the increases of VPD? Such changes would enhance solar radiation and evaporative demand, resulting in warmer and drier conditions, and thus higher in VPD (21, 45). We note, however, that an increase in downward surface solar radiation is mostly confined in the coastal states (California, Oregon, and Washington) and to northern Idaho and southwestern Arizona, whereas the decreases of Normalized Difference Vegetation Index (NDVI) are confined to Southern California and southwestern Arizona (*SI Appendix*, Fig. S5 *C* and *D*). These changes cannot explain a widespread increase of VPD′ and residual VPD′ across the entire WUS (*SI Appendix*, Fig. S5 *A* and *B*). Only southwestern Arizona exhibits both an increase in downward solar radiation and reduced NDVI; in this particular region, therefore, trends in both factors could contribute to the strong increase in observed VPD′.

**Fig. 5.**
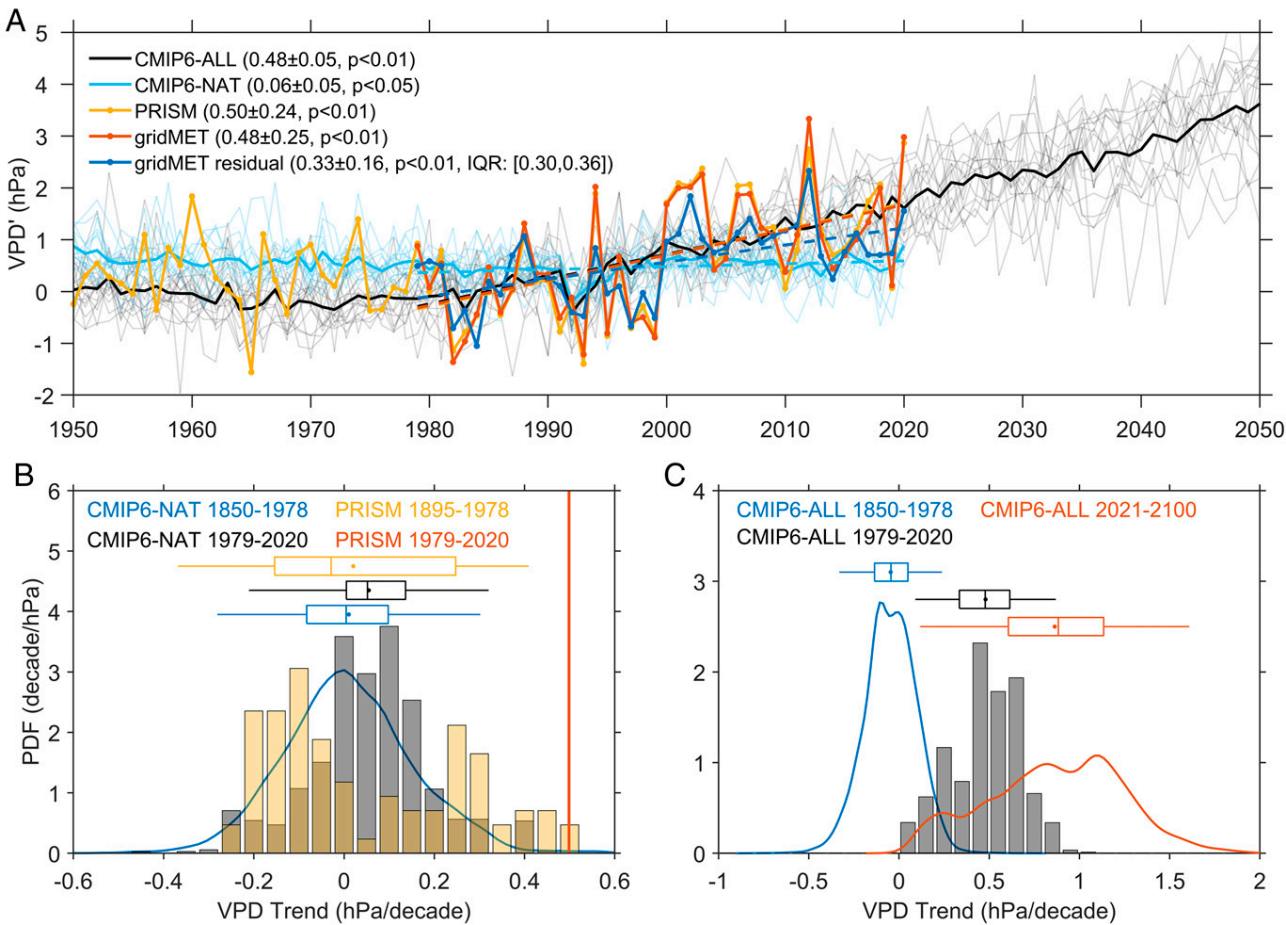
(*A*) Warm season mean VPD′ time series averaged over the WUS region and the trends during 1979 to 2020 calculated with climate models and observations (daily gridMET and monthly PRISM). The orange and blue line represents observed and residual VPD′ from gridMET, respectively; the yellow line represents observation from PRISM (a longer term monthly observational dataset covering 1895 to present that gridMET is based on); the black and cyan solid lines represent CMIP6-ALL and CMIP6-NAT simulations, and the thin gray and cyan lines are for all ensemble members from CMIP6-ALL and CMIP6-NAT, respectively. For the purposes of visual display, the VPD′ lines for ALL, NAT, and PRISM are forced to have the same mean value during 1979 to 2010 as gridMET. The VPD trends, 95% CI, and IQR (only for residual VPD′) labeled in the *Upper Left* corner are calculated for the 1979 to 2020 period. (*B*) PDF of VPD trend for PRISM observations and CMIP6-NAT; the vertical lines, dots, and whiskers for the box plots are defined as in Fig. 3*B*; VPD trend is calculated for every consecutive 42-y period within the periods listed above. (*C*) Same as *B* but for CMIP6-ALL. When calculating the ensemble-mean VPD trends and their PDFs in the CMIP6 simulations, VPD trend is first calculated for each ensemble member of each model, and weights are given to all the members in a way that all members from the same model are equally weighted and all models are also equally weighted.

The previous discussion and figures focused solely on the observations. We attempted to partition observed VPD trends into a component associated with circulation changes and a residual component likely to be dominated by the response of VPD to external forcing. In the following, we consider VPD trends in the CMIP6 models. Fig. 5*A* compares the VPD′ trend between models and observations over the same 1979 to 2020 period. Simulations with combined natural and anthropogenic forcings (which comprise historical simulations up to 2014 and the Shared Socioeconomic Pathway 5 - Representative Concentration Pathway 8.5 [SSP5-8.5] scenario integrations thereafter) show a significant (*P* < 0.01) warm season mean VPD trend of 0.48 ± 0.05 hPa/decade (Fig. 5 *A* and *C*), which is very similar to the observed VPD trend over the WUS.

In contrast, historical runs with only natural solar and volcanic external forcings show a very small mean VPD trend of 0.06 ± 0.05 hPa/decade (*P* < 0.05), with a middle 99% range from −0.37 to 0.46 hPa/decade (Fig. 5*B*). The observed VPD trend exceeds over 99% of the trend values that can be explained by natural climate forcings (solar and volcanic) and internal variability. Our natural variability estimates are based on a large number (∼15,000) of 42-y trend samples from 14 different climate models. The observed VPD trend is very similar to the mean of the modeled trends in the CMIP6 simulations with combined natural and anthropogenic external forcings. Over 1979 to 2020, the PDF of model VPD trends under “all forcings” spans the range from 0 to 1 hPa/decade (Fig. 5*C*). This range arises from natural climate variability, from model differences in historical external forcing, and from model differences in the response to forcing.

The difference between the multimodel ensemble with combined anthropogenic and natural forcings and the multimodel ensemble with natural forcing only—which we refer to as “ALL” and “NAT” hereafter—is widely used for estimating the anthropogenically forced component of climate change (28, 46–49). Here, differencing the means of the ALL and NAT multimodel ensembles yields an anthropogenically forced VPD trend of 0.42 hPa/decade, equivalent to 88% of the observed VPD trend over the period of 1979 to 2020. This is roughly 30% larger than our observationally derived residual VPD′ trend of 0.33 ± 0.16 hPa/decade.

## Discussion and Implications

The key part of our study uses observed atmospheric circulation patterns to quantify the contributions of different processes on the historical changes of climate conditions (50–53). It is likely that circulation patterns are influenced by global scale and expansion of the troposphere (49, 54). Our analogue method estimates the internally generated component of the observed VPD′ trend after the removal of the impact of thermal expansion of the troposphere on Z500. This does not mean, however, that the analogue VPD′ trend associated with variations in atmospheric circulation patterns is attributable to natural internal variability alone. Anthropogenic forcing can modulate the behavior of modes of atmospheric variability (55) and can affect the statistical properties of heatwaves and other extreme events (56–58).

One possible example of such modulation is a change in the frequency, intensity, and duration of East Pacific and West Coast ridging patterns that favor drought and fire weather condition over the WUS (59–61). A cluster analysis of Z500′ patterns (*SI Appendix*, Fig. S6) confirms that the frequency of occurrence of this ridging pattern has increased significantly from 10.2% during the period of 1979 to 2000 to 12.6% during the period of 2001 to 2020 (*P* < 0.1 as determined by a Monte Carlo test; *SI Appendix*, Supplementary Text). The intensity and duration of the pattern do not show significant changes (*SI Appendix*, Fig. S7). Our current analogue approach cannot determine if such pattern frequency changes are due to natural variability or are anthropogenically forced. If the latter is the case, they would not be removed in our analogue analysis and would be aliased in our estimate of natural variability. Such forced modulation of internal variability can therefore introduce biases in quantifying the internally generated component of the observed VPD trend. Consequently, our analogue-based estimate of the anthropogenic influence on the observed increase in fire weather risk is likely to be conservative—a conclusion that is supported by the larger anthropogenically induced VPD trend inferred from the CMIP6 simulations.

In contrast, the CMIP6 multimodel ALL and NAT ensemble means provide an estimate of forced VPD changes that has minimal contribution from internal variability. This is because the ALL and NAT ensemble means are calculated using coupled model simulations, which have random phasing of internal variability. Averaging over many different realizations of the 1979 to 2020 period (each with a different sequence of internal variability), and then averaging over different models, damps the “noise” of internal variability, yielding a clearer estimate of the externally forced signal. It is important, therefore, to recognize that the effects of natural variability must be accounted for in comparing the single (noisy) realization of the observations with a smoothed, “noise-reduced” multimodel average (62).

Natural internal variability is not the only factor contributing to the spread in VPD trends in ALL and NAT (Fig. 5 *B* and *C*). This spread also arises from intermodel differences in the applied forcings and in the climate responses to those forcings. Because of this uncertainty in the ALL and NAT VPD signals, the upper bound of the anthropogenically induced contribution to the VPD trend could be up to two times larger than the bound estimated by differencing the ensemble-mean VPD trends in the ALL and NAT simulations.

## Conclusions

Overall, we find that over the period 1979 to 2020, anthropogenic warming has contributed at least twice as much as natural variability to the rapid increase of fire weather risk. Our observational analogue-based attribution approach complements the estimates we obtain from global climate model simulations (10, 16, 28). Both methods constrain the range of the true contribution of anthropogenic forcing to the observed increase of VPD over the WUS. We estimate this range to be 0.33 to 0.42 hPa/decade or 68 to 88% of the observed trend. We have shown here that VPD is a robust, physically meaningful proxy for fire risk. During two specific extreme events—the August Complex fire and the California Creek fire in 2020—VPD values exceeded the highest values observed previously for similar atmospheric circulation patterns. For the August Complex “Gigafire” in the WUS, anthropogenic warming likely explains 50% of the unprecedented high VPD anomalies in the month of the fire’s occurrence (August 2020). On the August 16, 2020 start date of the August Complex fire and the September 4, 2020 start date of the California Creek fire, anthropogenic forcing likely contributed 32 and 52%, respectively, to the unprecedented high VPD′ at the beginning of these two extreme fire events.

Our results suggest that the WUS appears to have passed a critical threshold and that the dominant control on the fire weather variation in the WUS has changed from natural climate variability to anthropogenically forced warming. While natural climate variability can still significantly modulate the interannual to decadal variations of fire weather risk, the trend toward increasing risk will likely continue over the WUS. This change in risk requires urgent and effective societal adaptation and mitigation responses.

## Methods

### Trend Analysis.

Daily VPD, $e_{s}$, and $e_{a}$ were calculated (*SI Appendix*, Supplementary Text) using the gridMET dataset (41). For trend analysis, daily VPD, $e_{s}$, and $e_{a}$ during the warm season were averaged for each year before computing trends. Linear trends were calculated at each grid point and for the average values over each state of the WUS and over the entire WUS. Trends were estimated with three different methods: ordinary least squares regression (OLS), the nonparametric Theil–Sen estimator (63, 64), and Siegel’s repeated median estimator (65). All trend calculations were for the period of 1979 to 2020. Only trend results from OLS are listed in the main text; results from the other two methods are listed in *SI Appendix*, Table S3. The choice of trend estimator has minimal impact on our results.

### Ensemble Constructed Flow Analogue.

Our modified constructed analogue method is based on the flow analogue approach for daily data (38, 39) and the constructed analogue approach previously used for monthly data (40, 43). The basic strategy is to estimate the VPD′ of a given day associated with a specific atmospheric circulation condition. This estimate is obtained by using days with similar circulation conditions in other years during a reference period. There are three main steps. First, for each day in one particular analysis year, analogue days with similar circulation conditions are selected. Selection involves minimizing a distance function between daily standardized *Z*500′ fields in the North American and Northeastern Pacific domain during a reference period (here, we simply use the climatological period 1979 to 2010) in a 61-d window centered on the given day but not in the same year as the day we examine (66). Second, the top *N* number of the Z500′ analogue patterns with the smallest distance function are linearly combined to form a “constructed analogue” pattern that closely resembles the pattern we examine. In the third step, the *N* coefficients from the linear combination are applied to VPD′ on the *N* analogue days to form an analogue VPD′.

The original flow analogue method proposed by Yiou et al. (38) used the median value of the daily VPDs on the *N* analogue days to represent the contribution from circulation patterns; our experiments show that using a “constructed analogue” approach similar to those employed for dynamical adjustment with monthly data (43) can increase the explained variance (*R*^2^) of the observed daily/seasonal VPD′. The “constructed analogue” approach yields a substantially larger analogue VPD′ trend than the “median analogue” (*SI Appendix*, Table S5). For the purposes of this study, we set the analogue number *N* to 20; other plausible choices do not impact the overall results (we tested *N* values from 5 to 60; reference *SI Appendix*, Table S5).

For classifying circulation types, we chose Z500 instead of sea level pressure (SLP). There were two reasons for this choice: 1) most of the WUS is above sea level, and 2) Z500 explains more of the spatiotemporal variance of surface temperature anomalies than SLP (39). As noted above, it is physically plausible that anthropogenic forcing could also change atmospheric circulation patterns on decadal scales (52, 67–69). To reduce the impact of anthropogenically forced global warming in our analysis, we subtract the daily global-mean Z500 value from the daily Z500 variation at each grid point (49, 70) before computing analogues. Sippel et al. (71) applied a high-pass filter to the Z500 data at each grid point to reduce circulation variability at the centennial time scale, under the assumption that the forced circulation trend was smooth and additive. This is not suitable for our study due to the relatively short observational record and the fact that it could remove some of the decadal natural variability.

Our approach removes the large-scale increase in Z500 due to expansion of the atmospheric column as global surface temperature increases (59). However, it does not remove any anthropogenically driven changes in atmospheric circulation patterns. If such changes exist (72), they will be incorrectly labeled as natural climate variability in our analogue analysis. In the case of such external modulation of internal variability patterns, our analysis will overestimate the impact of natural climate variability and so underestimate the contribution of anthropogenic forcings to the observed VPD changes.

Other studies have used an empirical orthogonal function (EOF) approach to estimate large-scale features of circulation patterns (38). There are two reasons why we do not employ an EOF approach here: 1) for extreme circulation conditions, for example, the condition shown in Fig. 3*C*, EOF-constructed Z500 has smaller maximum amplitude than the raw Z500, thus leading to underestimation of analogue VPD′; and 2) our experiments show that with 20 analogues, using EOF-constructed Z500 instead of raw Z500 does not improve the fitting skill of the analogue (*SI Appendix*, Table S6). To reduce high-frequency synoptic fluctuations, we simply apply a 5-d moving average filter (73, 74).

For the VPD data used to train the analogue model, we first apply a locally weighted scatter-plot smoother to fit a locally weighted quadratic polynomial with a span value of 0.75. The rationale for this fitting procedure is to represent low-frequency anthropogenic influence on VPD. The local quadratic fit is then removed from the raw VPD data before calculating the VPD anomalies. Sensitivity tests indicate that employing this fitting procedure increases the *R*^2^ by a small amount (on both daily and seasonal timescales, about 2%) (*SI Appendix*, Table S7).

To explore the sensitivity of the analogue results by use of different plausible data selection and processing choices, we generate 180 different analogue schemes. These are based on all possible combinations of the following four sets of selection options: 1) four reanalysis datasets for estimating Z500, including the fifth generation of the European Centre for Medium-Range Weather Forecasts (ECMWF) atmospheric reanalysis (ERA5), the Modern Era Retrospective analysis for Research and Applications version 2 (MERRA-2), the National Centers for Environmental Prediction (NCEP) Climate Forecast System Reanalysis (CFSR), and the Japanese 55-y Reanalysis (JRA55); 2) three distance functions (Euclidean distance, Pearson’s correlation, and Spearman’s rank correlation); 3) three spatial domains for Z500 (160° to 80°W, 20° to 60°N; 150° to 90°W, 25° to 55°N; and 140° to 100°W, 30° to 50°N); and 4) to avoid selecting consecutive days from the same weather event, analogues are selected from every other 5 d. This yields a total of 4 × 3 × 3 × 5 = 180 analogue schemes; their mean analogue VPD′ is used to represent the overall analogue contribution to the VPD′ trend and their IQR to describe the distribution of the analogue (see the IQR results in Fig. 4 *C*–*E*). The impact of using different reanalysis datasets, distance functions, and domains for Z500 is shown in *SI Appendix*, Fig. S8. Overall, using an average of the 180 schemes improved the fitting skill (*R*^2^) and thus led to a more robust result. Importantly, it allowed us to explicitly quantify uncertainty in the contribution of internally generated VPD changes to observed VPD trends.

## Data Availability

All data used in this study are publicly accessible. Data access links are included in *SI Appendix*.
